# Investigation of the Syncytial Nature of Detrusor Smooth Muscle as a Determinant of Action Potential Shape

**DOI:** 10.3389/fphys.2018.01300

**Published:** 2018-09-20

**Authors:** Shailesh Appukuttan, Mithun Padmakumar, John S. Young, Keith L. Brain, Rohit Manchanda

**Affiliations:** ^1^Computational Neurophysiology Lab, Department of Biosciences and Bioengineering, Indian Institute of Technology Bombay, Mumbai, India; ^2^School of Pharmacy and Biomedical Sciences, University of Portsmouth, Portsmouth, United Kingdom; ^3^Institute of Clinical Sciences, College of Medical and Dental Sciences, University of Birmingham, Birmingham, United Kingdom; ^4^Christ Church, University of Oxford, Oxford, United Kingdom

**Keywords:** urinary bladder, smooth muscle, detrusor, syncytium, action potential shape, feature correlation, neuron model

## Abstract

Unlike most excitable cells, certain syncytial smooth muscle cells are known to exhibit spontaneous action potentials of varying shapes and sizes. These differences in shape are observed even in electrophysiological recordings obtained from a single cell. The origin and physiological relevance of this phenomenon are currently unclear. The study presented here aims to test the hypothesis that the syncytial nature of the detrusor smooth muscle tissue contributes to the variations in the action potential profile by influencing the superposition of the passive and active signals. Data extracted from experimental recordings have been compared with those obtained through simulations. The feature correlation studies on action potentials obtained from the experimental recordings suggest the underlying presence of passive signals, called spontaneous excitatory junction potentials (sEJPs). Through simulations, we are able to demonstrate that the syncytial organization of the cells, and the variable superposition of the sEJPs with the “native action potential”, contribute to the diversity in the action potential profiles exhibited. It could also be inferred that the fraction of the propagated action potentials is very low in the detrusor. It is proposed that objective measurements of spontaneous action potential profiles can lead to a better understanding of bladder physiology and pathology.

## 1. Introduction

Excitable cells exhibit a variety of action potential (AP) shapes but, generally, individual cells from the same tissue, or region of tissue, tend to display a common AP shape, characteristic of that cell type. The smooth muscle layer of the urinary bladder wall (detrusor) contrasts in this regard; it is found to exhibit APs of widely varying temporal profiles (Meng et al., 2008). Remarkably, these variations in AP shapes are observed even in electrophysiological recordings obtained from a single cell. The physiological basis for this diversity is currently not understood, and calls for a deeper analysis, as it could pave the way toward a better understanding of bladder electrophysiology and its contribution to downstream events, e.g., Ca^2+^ dynamics and contraction. Intuitively, there could be only two possibilities for obtaining APs of different profiles: (i) multiple AP generation mechanisms, i.e., differences in ion channel compositions between cells, or (ii) a single AP generation mechanism with variable modulation due to factors linked to syncytial function, including innervation. A preliminary study demonstrated the origin of different AP shapes from a single basic underlying mechanism (Appukuttan et al., 2015a).

Generally, the focus of investigation when analyzing APs principally lies in the frequency of spiking, and the characteristics of the individual APs are often overlooked. Both are emergent properties of the tissue depending upon a wide range of parameters and mechanisms, and in the case of detrusor smooth muscle (DSM), also involving the complex cellular environment. Changes in the underlying factors could potentially result in pathology such as the overactive bladder (Fry et al., 2002). Though analysis of AP frequency could assist in identifying abnormalities arising in pathology, we believe that the individual AP features potentially offer greater insight into changes in any of the underlying factors, such as ionic mechanisms and syncytial interactions.

Prior studies have shown that the DSM cells are electrically coupled to one another via low-resistance electrical pathways formed by gap junctions, thereby resulting in a three-dimensional electrical syncytium (Fry et al., 2004). This implies that the electrical activity recorded from a particular cell in the syncytium need not necessarily have been initiated in that cell, but may have originated at any other cell within its electrical “reach”. This also entails that the response of the cell is determined not just by the biophysical properties of that particular cell, but also by the properties and location of other cells in the syncytium. Past studies have demonstrated that the excitability of individually identical cells differ based on the size of the syncytium, their location within it, and the extent of gap junctional coupling between neighboring cells (Appukuttan et al., 2017a). Further, the electrical syncytium itself can be partitioned into sub-syncytia, representing smaller muscle bundles that are internally well-coupled, whereas coupling between bundles is comparatively poor, as reported for the detrusor (Bramich and Brading, 1996) and colonic smooth muscle (Spencer et al., 2002). The pattern of innervation further complicates the biophysical setting. In the case of the detrusor, a single nerve terminal could innervate multiple DSM cells and each DSM cell could potentially receive inputs from multiple nerve terminals, resulting in a complex many-to-many mapping between muscle and nerve. These considerations make the interpretation of DSM electrophysiological signals problematic.

In view of the above, a computational approach might assist in unraveling how DSM action potential profiles are determined. We employed a previously established passive model of the detrusor, developed using the compartmental modeling technique (Appukuttan et al., 2015b), and incorporated an identical action potential generation mechanism in each DSM cell in order to study the initiation, propagation, and modulation of AP shapes in the detrusor. The detrusor has not been found to follow any single AP template, and the DSM cells are known to possess an array of nine or more active channels contributing to their generation (Brading, 2006; Steers and Tuttle, 2009; Brading and Brain, 2011; Petkov, 2011). Models for the detrusor specific AP that satisfactorily emulate this ensemble of ion channels are yet to be developed. The purpose of the present study has been to obtain a better understanding of their syncytial nature and gather insights into their role toward the diversity in AP shapes. It was deemed appropriate to employ a “standard” AP shape for such an investigation so as to delineate the contributions arising from the syncytial organization of the cells. The classical Hodgkin-Huxley (HH) model, being a well–understood AP model, was therefore employed to explore this computationally.

The urinary bladder regulates both the storage and voiding of urine. Localized contractions help the bladder maintain tone during the storage phase as the bladder increases in volume with the accumulation of urine (Drake et al., 2003; Andersson, 2010, 2011). Whereas, coordinated contractions along the bladder wall, along with relaxation of the urethra, result in emptying the bladder during the voiding phase. Experimental studies in the past have suggested that spontaneous spiking of the cells causes the localized contractions, while the coordinated contractions are the concerted outcome of nerve-evoked APs (Andersson, 2011). Overactive bladder is an example of bladder pathology, more prevalent amongst the elderly population, which severely debilitates the affected. It is characterized by a frequent urge to urinate, with often an inability to control the urge, thereby hampering lifestyle (Milsom et al., 2001; Chapple et al., 2005). The etiology is often associated with detrusor overactivity wherein the detrusor undergoes unsolicited contractions arising from aberrant spiking. We believe that the undesirable spread of originally localized contractions, via the propagation of spontaneous APs owing to changes in the underlying syncytial features, could play an important role under such pathlogical conditions.

The detrusor is known to exhibit spontaneously occurring action potentials (Meng et al., 2008). The spontaneity of the action potentials is believed to have both a myogenic and neurogenic origin. While non-smooth muscle pace-making cells, such as Interstitial Cells (ICs), present in the urinary bladder wall are reported to be responsible for myogenic APs (McCloskey and Gurney, 2002; Hashitani et al., 2004; Kubota et al., 2006; Shen et al., 2008), the spontaneous asynchronous release of neurotransmitter packets from the nerve terminals causes the neurogenic APs (Young et al., 2008). These monoquantal events are termed as spontaneous excitatory junction potentials (sEJPs) as they are not elicited by an AP on the presynaptic nerve terminal. This is evidenced by the inability to abolish them using TTX, a blocker of Na^+^ channels (Young et al., 2008). This is in contrast to excitatory junction potentials (EJPs) which involve the synchronous release of neurotransmitter packets from the nerve terminals following presynaptic activation, and are thereby affected by TTX. These underlying differences translate to differences in the evoked postsynaptic potentials, with the sEJPs reflecting very rapid spatial and temporal decays in comparison to EJPs (Tomita, 1967; Appukuttan et al., 2015b). It is pertinent to highlight that the above does not hold true for sEJPs in the intestinal smooth muscle, which differ from other autonomic neuromuscular junctions via their sensitivity to TTX, wider spatial spread and being produced from the synchronous activation of a population of enteric motor neurons (Spencer et al., 2001).

**Figure 1:**
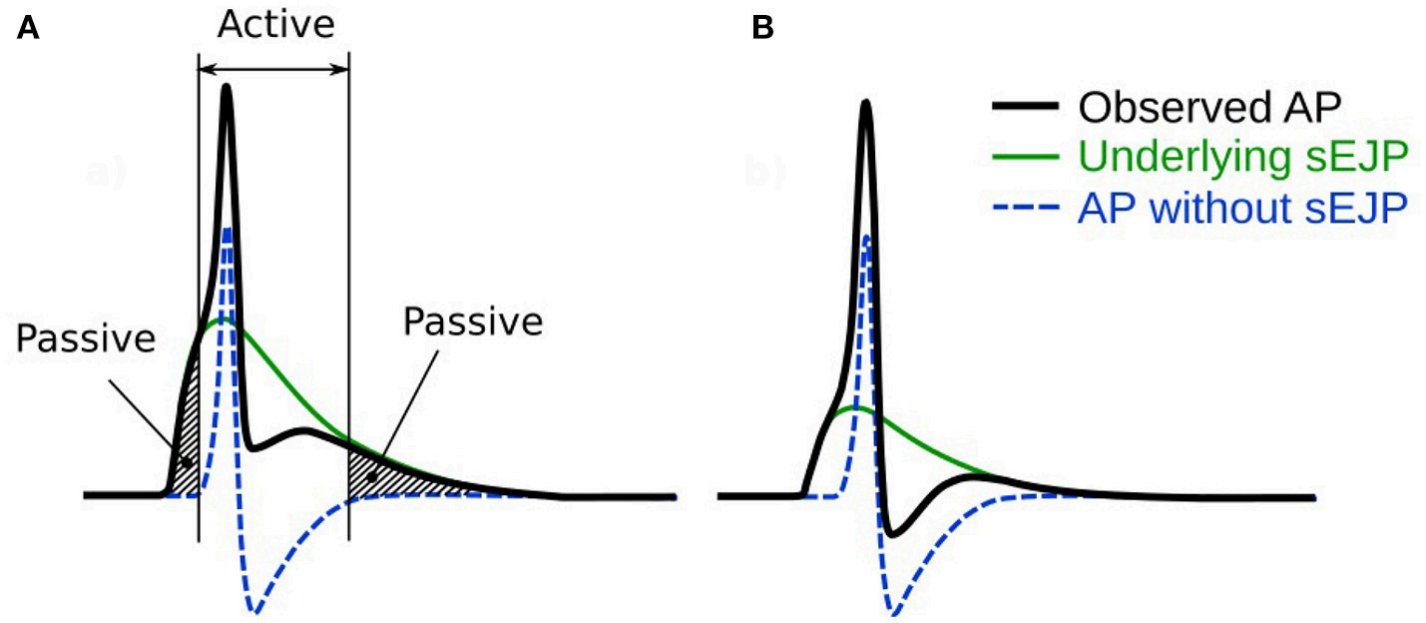
An illustration of our hypothesis for the generation of APs. It is hypothesized that the convex-upward foot and the after-depolarization (ADP) are caused by the presence of an underlying spontaneous excitatory junction potential (sEJP, green curve) which initiates the AP (black curve). The dashed line represents the shape of the AP that would have been generated solely by the ion channel mechanisms if the underlying sEJP was absent. It can be observed that a larger underlying sEJP **(A)** would result in lowered foot convexity with a larger ADP and reduced AHP, compared to that of the APs with smaller underlying sEJP **(B)**. The passive and active regions of the AP are indicated in **(A)**.

We have in the past proposed that the diversity in AP profiles arises predominantly from varying levels of superposition of a more or less stereotypical “native” AP with a second more variable component. The latter being contributed by junction potentials with a broad range of amplitudes and dynamics (Padmakumar et al., 2012), as illustrated in Figure 1. A similar observation was made earlier in skeletal muscles by Fatt and Katz (1951). They have shown that the shape of an AP changes when it propagates away from the site of initiation (see section 4), with the foot of the action potential notably changing from convex-upwards to concave-upwards. As smooth muscle cells form electrical syncytia, it is probable that an AP generated at one cell could assume different shapes as it propagates to other cells. In the present work, we test this hypothesis by comparing results obtained from experiments and simulations. Accordingly, the focus of this study has been restricted to the subset of APs with a neurogenic basis. It is pertinent to emphasize that the aim of the work presented here is to demonstrate that syncytial interactions can be an important factor for AP diversity, and that some potential insights could be availed by analyzing the individual AP profiles. We do not seek to dismiss other possible factors leading to AP diversity, such as variations in ionic channel compositions and/or other mechanisms. This study is particularly important in view of the diversity in AP profiles observed even from a single cell during a continuous recording, without external interventions.

## 2. Methods

### 2.1. Electrophysiological recordings

Mice of the C57BL/6 strain, of either gender, weighing 18–30 g, were sacrificed by head concussion followed by cervical dislocation. Efforts were undertaken to minimize the number of animals used and their suffering. After removing the urinary bladder, the connective tissue surrounding the bladder was withdrawn, while the urothelium was left intact. The ventral wall of the bladder was opened longitudinally from the neck (posterior) to the top of the dome (anterior). Tissue strips, containing a few bundles of smooth muscle, 3–4 mm long and 1–2 mm wide, were cut. Strips were pinned out on a Sylgard-lined plate at the bottom of a shallow chamber (volume, approximately 1 ml), which was mounted on the stage of an upright microscope. Preparations were superfused with warmed (35°C) physiological saline solution (PSS) (composition, mM: NaCl, 120; KCl, 5.9; MgCl_2_, 1.2; CaCl_2_ 2.5; NaHCO_3_, 15.5; NaH_2_PO_4_, 1.2 and glucose, 11.5; gassed with 95% O_2_ and 5% CO_2_) at a constant flow rate (100 ml/h), maintaining a pH of 7.2–7.3 (Hashitani and Brading, 2003)

Preparations, once pinned, were allowed to equilibrate for a minimum of 30 min before initiating electrophysiological recordings. Individual DSM cells in muscle bundles were impaled with glass capillary microelectrodes, filled with 0.5 M KCl (tip resistance, 100–300 MΩ). Changes in membrane potential were recorded using a high input impedance amplifier (Axoclamp-2B, Axon Instruments, Inc., Sunnyvale, CA, USA), digitized using PowerLab/4SP (ADInstruments, Chalgrove, UK) at either 1 or 4 kHz, and stored on computer for later analysis.

### 2.2. Model development

A previously established three-dimensional passive model of smooth muscle syncytium (Appukuttan et al., 2015b) was adopted and extended for this study. Modeling was undertaken employing the compartmental modeling technique on the NEURON simulation environment (Carnevale and Hines, 2006). Development of the syncytial model primarily involved the following steps: (i) development of a template model for individual DSM cells, (ii) designing a gap junctional coupling mechanism to electrically connect two cells, and (iii) building the syncytial topology by creating multiple cells using the template, positioning these in three-dimensional space, and coupling adjacent cells by means of the gap junction mechanism. Table S1 summarizes the important model parameters. Individual detrusor smooth muscle cells are fusiform in shape, but were approximated to be cylindrical with a long and narrow profile: 200 μm in length and 6 μm in diameter (Fry et al., 1999; Appukuttan et al., 2015b). The number of compartments (segments) per cell was kept high (nseg = 51) to benefit from high spatial resolution within each cell, leading to each segment being <4 μm. Such a resolution permitted localized stimulation of individual cells.

The original syncytial model was purely passive, with the cells possessing no active ion channel mechanisms, and was used to investigate passive electrical properties of the DSM syncytium (Appukuttan et al., 2015b). As the present study aims to explore the diversity in AP profiles in syncytium, we augmented the syncytial model by endowing every DSM cell with the HH set of channels so as to enable the generation of action potentials. Adjacent cells were coupled by means of resistive pathways representing gap junctions which allowed bidirectional flow of current between the coupled cells. The magnitude and direction of gap junctional current was determined by the potential gradient between the coupled cells, and the extent of coupling. This is illustrated in Figure S1A. The gap junctional resistance, in the standard model, was kept as 30.6 MΩ (32.68 nS); but this has been varied in certain simulation settings and described accordingly. The cells were connected to form a cubic lattice arrangement of cells, thereby forming a three-dimensional syncytium (see Figure S1B). Under such a topology, each cell in the interior of the syncytium (i.e., non-peripheral) is electrically coupled to six other adjacent cells, two along each axis. Experimental findings have revealed functional syncytia to contain up to 100 cells (Neuhaus et al., 2002). To approximate this, the size of the syncytial model was set as 5-cube across all simulation scenarios, unless otherwise specified. Cells were stimulated by means of synaptic inputs, with the location of the stimulus being varied across the simulations. Synaptic activity was simulated by means of an AlphaSynapse in NEURON, which produces a localized conductance change in the membrane using an alpha function (Carnevale and Hines, 2006; Appukuttan et al., 2015b). The reversal potential of the synapse was kept at the default value of 0 mV. This injects inward current to the stimulated cell, except when the membrane potential overshoots 0 mV during an AP. The time constant of synaptic input was set to 10 ms, which is within the experimentally reported range of 5–89 ms (Young et al., 2008). We chose a value closer to the lower bounds in accordance with the shorter width of HH APs, thereby ensuring that an sEJP would not overly dominate the AP profile. Peak synaptic conductance, in the standard model, was adjusted to the minimal supra-threshold stimulus corresponding to the centroidal cell in the syncytium; the centroidal cell being the least excitable owing to its location. In certain simulation settings, the synaptic peak conductance was varied, and these changes have been highlighted wherever applicable.

### 2.3. Measurement of correlation

Pearson's correlation coefficient (*r*) was evaluated to test the correlation between any two parameters of interest. In case of experimental recordings, the *r*-value was calculated for individual cells, and the confidence of the correlation trends are measured using Student's one sample *t*-test.

### 2.4. AP features and their measurements

**Figure 2:**
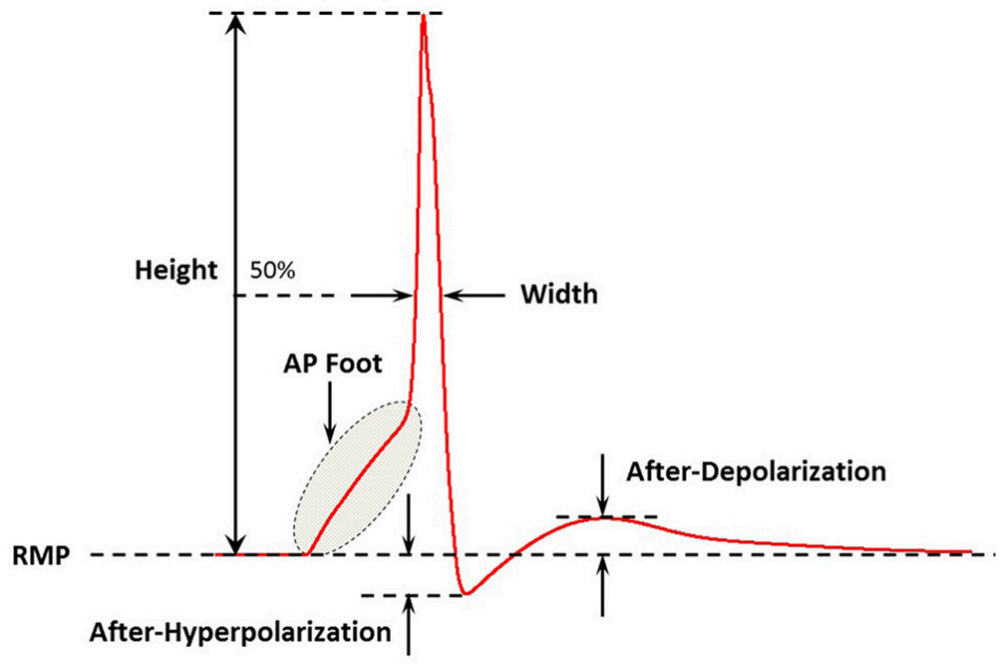
A schematic figure showing the key features of the action potential profile which were used in the study. Adapted from Appukuttan et al. (2015a).

APs of a wide variety of shapes and sizes are discussed in the present study. It is imperative to quantifiably describe these AP profiles in order to distinguish and differentiate between them. To accomplish this, we follow the methodology adopted previously (Appukuttan et al., 2015a) of evaluating the following five features for each AP shape, as illustrated in Figure 2:

- *Height*: Maximum membrane depolarization (in mV) measured from the resting membrane potential (RMP) to the peak of the AP
- *Width (Full Width at Half Maximum)*: Time (in ms) measured between the crossing of half the AP height across the rising and falling phases of the AP
- *Convexity*: Provides a quantitative measure of the AP foot convexity; the foot of the AP being defined as the phase from the initiation of membrane depolarization to the AP threshold. The foot contributes in moving *V*_*m*_ from its resting level toward the threshold
- *After-Hyperpolarization (AHP)*: Difference (in mV) measured between the RMP and the first local minimum following the AP peak
- *After-Depolarization (ADP)*: Difference (in mV) measured between the first local maximum following the AHP and the RMP

**Figure 3:**
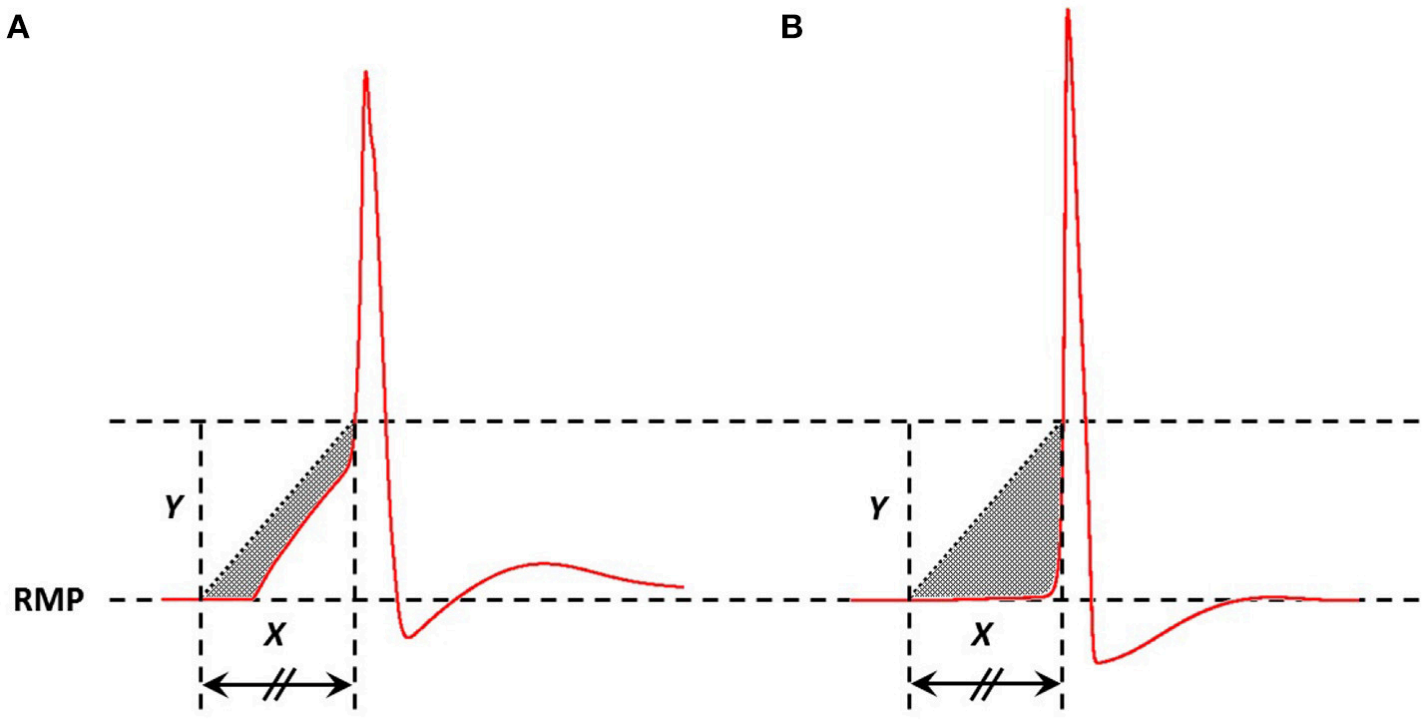
Method adopted to quantify convexity of AP foot. The shaded area indicates the area between the AP curve and the straight line joining the two pre-defined points. Area below the straight line is considered as negative area and that above, if present, as positive (absent in above cases). Here, the signal in **(A)** has a greater value of convexity than that in **(B)**.

In a previous study, we have demonstrated that an appropriate technique to measure the AP foot convexity involves evaluation of the area enclosed between a pre-defined line and the AP foot (Appukuttan et al., 2017b). This technique is illustrated in Figure 3. The measure thus obtained is denoted as *C*_*X, Y*_ indicating the measure of AP foot Convexity evaluated for a depolarization of *Y* mV over a time frame of *X* ms, where *X* and *Y* define the line. The values of *X* and *Y* for this study were selected in accordance with the requirements of this method (Appukuttan et al., 2017b). This approach has been shown to be more applicable over other methods such as evaluation of the radius of curvature along the AP curve.

## 3. Results

**Figure 4:**
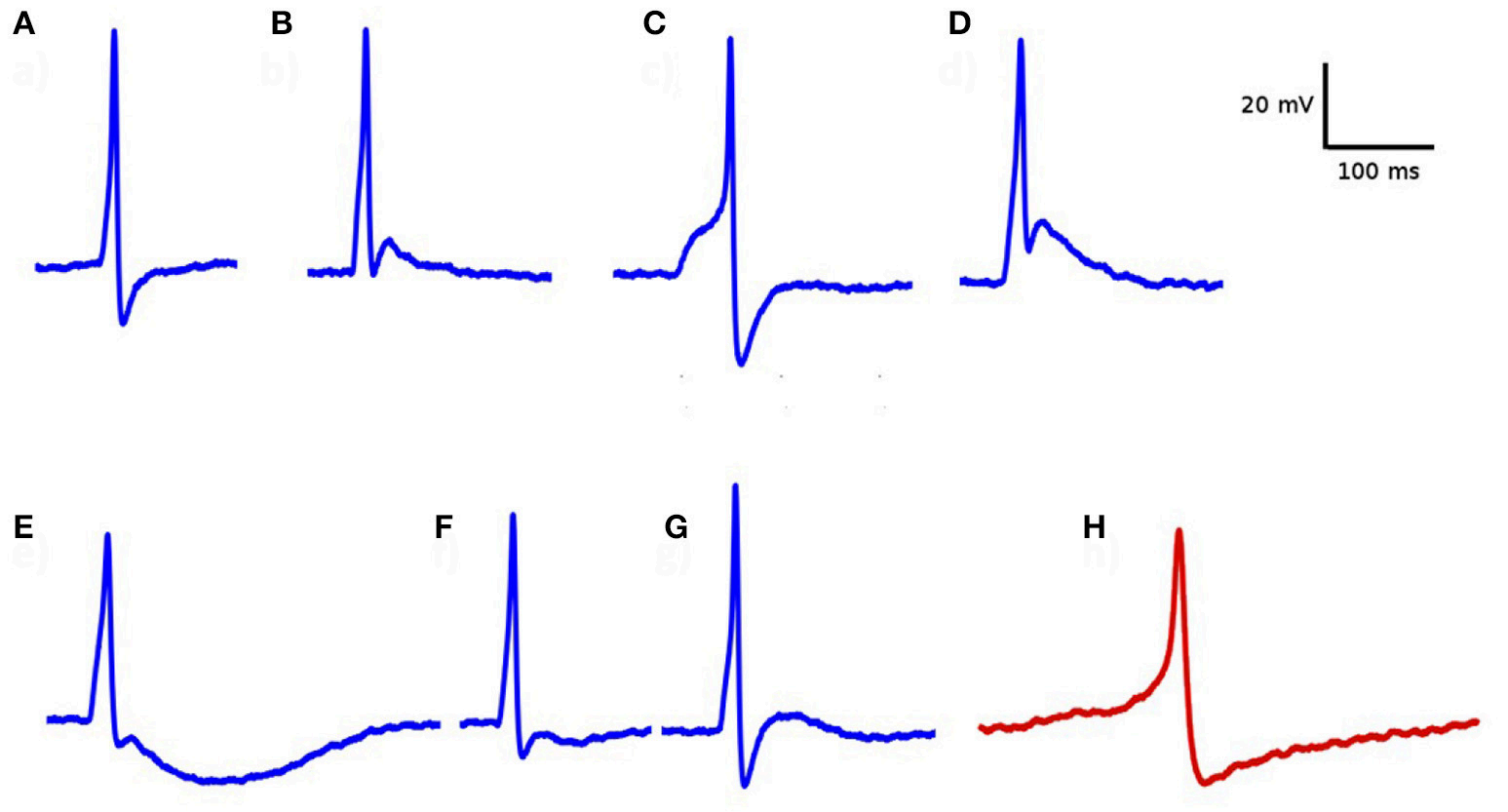
Diversity in action potential shapes observed in detrusor smooth muscle. **(A–G)** action potentials recorded from a single detrusor smooth muscle cell. All of these have convex-upward feet, indicating that they are of neurogenic origin. **(H)** An example of “pace-maker” type action potential, given for comparison. Note the ramp-type foot and the lack of after-depolarization. These types of APs are believed to have a myogenic origin.

Electrophysiological recordings from DSM cells demonstrated that important differences can be observed in the temporal profile of the individual APs. As illustrated in Figure 4, these variations include differences in their height, width, the shape of the AP foot and extent of hyperpolarization, and after-depolarization. The most common active signals observed in the DSM can very broadly be classified into two categories, as reported earlier (Meng et al., 2008; Hayase et al., 2009). The first type has a fast rising phase (“spike-type”) whereas the second type has a much slower ramp-like depolarization phase (“pacemaker-type”). For our study here, the action potentials of the pace-maker type were ignored as these are believed to originate from non-smooth muscle pace-making cells, perhaps the Interstitial Cells (ICs) (McCloskey and Gurney, 2002; Hashitani et al., 2004; Kubota et al., 2006; Shen et al., 2008). Our focus here lies in spontaneous action potentials of the spike-type—which is of neurogenic origin. Padmakumar et al. (2018) identified neurogenic and myogenic sAPs based on features evaluated from sAP profiles. For pooled data, they estimated that around 83% of the sAPs were of neurogenic origin. But the relative contributions from neurogenic and myogenic origins could vary significantly for individual cells.

### 3.1. Analysis of electrophysiological recordings

**Figure 5:**
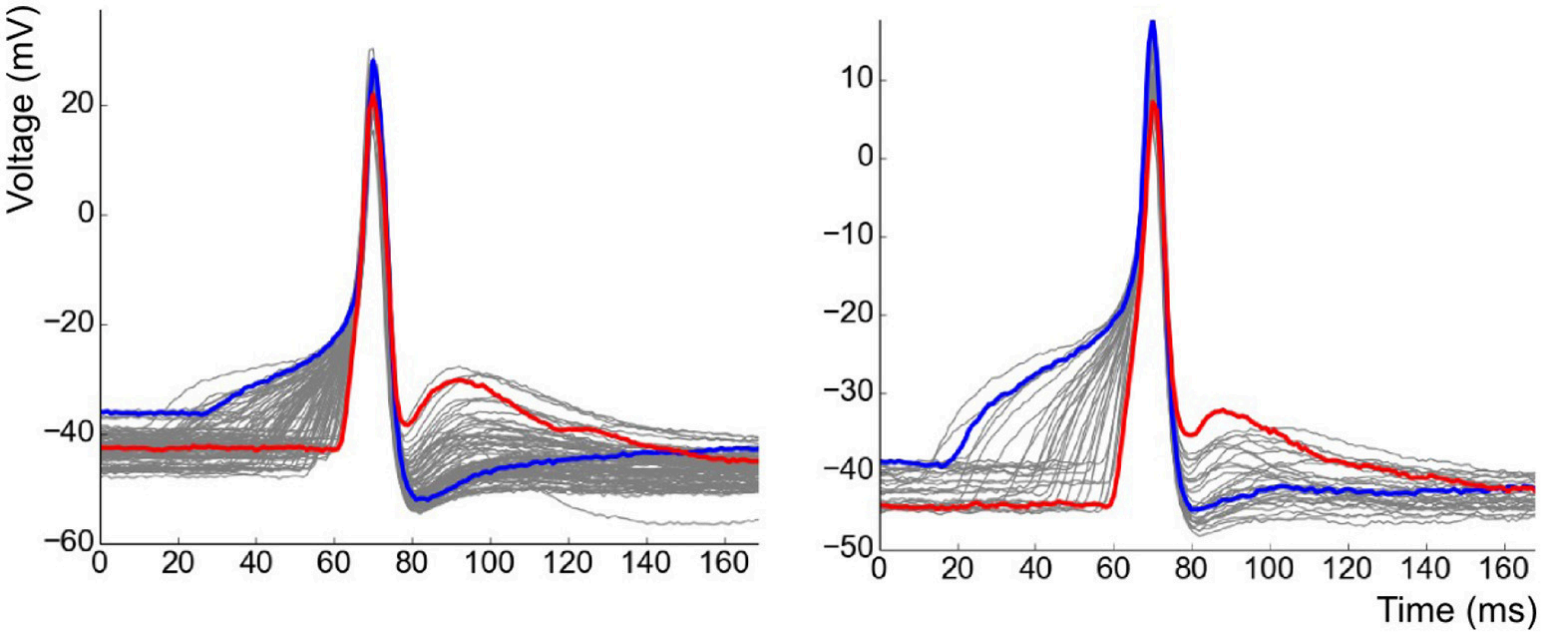
Collection of all APs observed in two of the cells superimposed over one another. Examples of strongly contrasting shapes have been highlighted in each case.

After having removed APs of the pacemaker type from the analysis, recordings containing a minimum of 30 APs were shortlisted. APs contaminated by the interference of other independent APs or sEJPs were also removed from the study. This resulted in a collection of 11 cells from 6 animals with a total of 712 APs recorded in them. The overlapped plots of the APs in two of the cells are shown in Figure 5. Note the striking correlation of the foot and tail features of the two highlighted APs. The AP with a wider, more convex foot has a larger AHP and smaller ADP, and vice versa.

According to our hypothesis for diversity in AP shapes, every AP with a convex foot will have an underlying sEJP that elicited it (Figure 1). This underlying sEJP, via superposition, accounts for the observed profiles of the passive and active regions of the corresponding AP. As seen in Figure 1, the effect of sEJP would be present in the foot as well as in the tail. This implies that there should be a correlation between the features of the foot and tail of the AP.

The features, previously described in section 2.4, were determined for each of the APs. Once the features were obtained, the Pearson correlation coefficients between the features were calculated. Correlations were deemed statistically significant if the 99% confidence interval excluded zero. Strong correlations were found to exist between: (i) Height and Width (−0.63±0.29; *P* < 0.01), (ii) Convexity and AHP (0.74±0.08; *P* < 0.01), (iii) Convexity and ADP (−0.63±0.16; *P* < 0.01), and (iv) AHP and ADP (−0.77±0.14; *P* < 0.01). These correlations for all the 11 cells are shown in Table 1.

**Table 1:** Correlations observed between the features measured from experimentally recorded data for 11 cells.

**Cell No**.	**Height vs. Width**	**C_25,30_ vs. AHP**	**C_25,30_ vs. ADP**	**AHP vs. ADP**
1	−0.54	0.81	−0.88	−0.95
2	−0.04	0.74	−0.75	−0.91
3	−0.62	0.70	−0.76	−0.89
4	−0.90	0.78	−0.64	−0.64
5	−0.78	0.83	−0.69	−0.75
6	−0.79	0.74	−0.46	−0.73
7	−0.86	0.86	−0.59	−0.62
8	−0.72	0.66	−0.75	−0.82
9	−0.80	0.63	−0.39	−0.51
10	−0.73	0.81	−0.57	−0.80
11	−0.13	0.63	−0.44	−0.83
Mean	−0.63	0.74	−0.63	−0.77
SD	0.29	0.08	0.16	0.14

*The mean and standard deviation of the correlations are also shown*.

**Figure 6:**
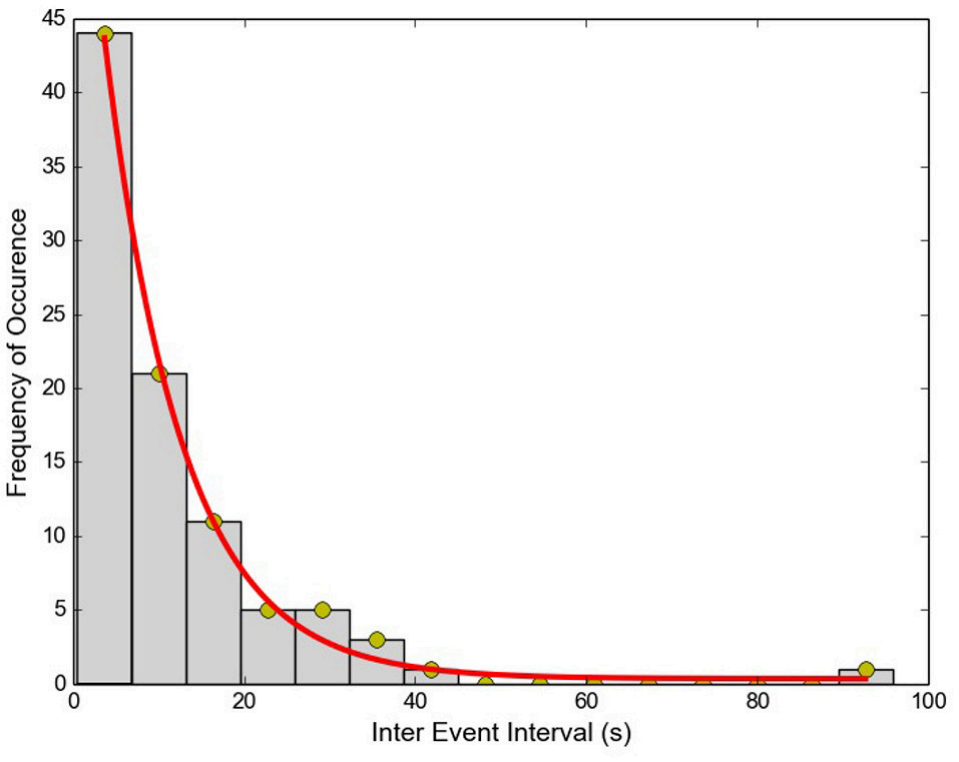
Inter-Event interval histogram for APs recorded from a single cell. Red line shows the exponential fit to data (*R*^2^ = 0.99).

The Inter-Event Interval (IEI) histogram was computed for the collection of APs from each of the cells. One such example is shown in Figure 6. For all 11 cells tested, the trend was not significantly different from a single exponential function (Kolmogorov–Smirnov test with *P* > 0.05 for each cell), signifying that the APs are independent, stochastic events. The occurrence of any AP does not influence the occurrence or non-occurrence of others APs.

### 3.2. Analysis of AP diversity in simulation studies

**Figure 7:**
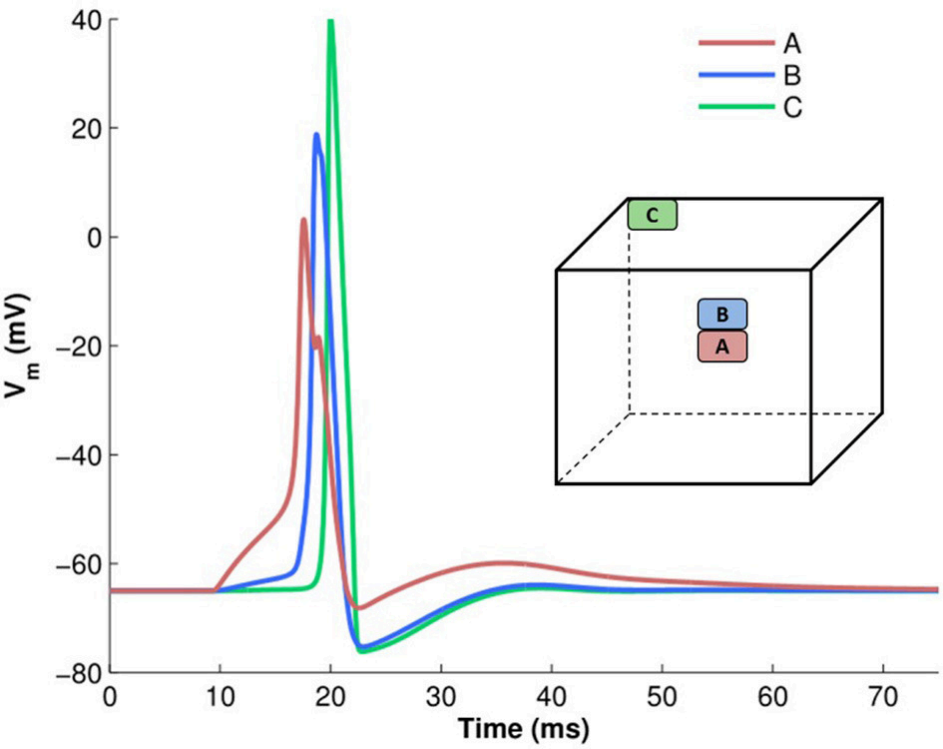
Examples of variety in AP shapes observed in a 5-cube syncytium when stimulated at the centroid cell. The cube illustrates the location from where each recording is obtained. The APs have been aligned at the instant of AP initiation.

To analyze action potential shapes computationally using our model, we (i) stimulated the centroid cell in our syncytium and recorded the APs generated at each cell in the syncytium for different sizes of syncytium, and (ii) stimulated each cell in a 5-cube syncytium, one after another, and recorded the APs recorded at specific locations within the syncytium. This was an extension of the simulations undertaken previously (Appukuttan et al., 2015a), with a requirement for more detailed analysis. It was observed that APs of a variety of shapes and sizes were produced in the model, with an evident gradation from one type to another. Figure 7 illustrates a few of these AP profiles recorded at different locations in the syncytium. The AP profiles were analyzed by quantifying the five features discussed in section 2.4. All five features were found to exhibit a notable spread across the ensemble of AP profiles. The AP height varied by over 35 mV (range: 68.2 to 105.4 mV) with APs recorded at the vertex cells being the largest, and reduction in the height when moving closer to the interior of the syncytium. The stimulated cells produced the APs with smallest heights, but with largest widths, as seen for cell A in Figure 7 (pink trace). Also, only these cells, and to an extent their immediately neighboring cells, produced APs with convex AP feet. The majority of the cells in the syncytium exhibited a more “concave-upwards” rise to the AP peak, as has been reported previously (Appukuttan et al., 2017b). This is seen for cell C in Figure 7 (green trace). The extent of after-hyperpolarization (AHP) and after-depolarization (ADP) also varied across the syncytium, depending on the syncytial location of the cell in which the AP was recorded. The stimulated cells, and its neighbors, produced APs with a lower level of AHP and a higher ADP, while the trend was opposite for the more distant cells.

From the above observations, it is discernible that the five AP features evaluated here share strong correlations. Tables 2, 3 summarize the correlation coefficients between the AP features under different settings. As expected from above, the AP height shares a strong negative correlation (−0.83 to −0.94) with the AP width. Similarly, a strong negative correlation (−0.95 to −1.00) exists between the extent of AHP and ADP. However, AP convexity shares a strong negative correlation (−0.66 to −0.93) with the extent of AHP, and a strong positive correlation (0.70 to 0.96) with the level of ADP.

**Table 2:** Correlation between AP features from simulations for syncytia of varying sizes, when stimulus is applied at the centroid cell and APs are recorded at every cell.

**Correlation**	**Stimulated at centroid, Syncytium size:**			
	**3-Cube**	**5-Cube**	**7-Cube**	**15-Cube**
Height vs. Width	−0.94	−0.92	−0.93	−0.93
*C*_25,10_ vs. AHP	−0.93	−0.88	−0.89	−0.76
*C*_25,10_ vs. ADP	0.96	0.91	0.90	0.85
AHP vs. ADP	−0.99	−1.00	−1.00	−0.95

**Table 3:** Correlation between AP features from simulations for a syncytium of size 5-cube, when stimulus is applied successively at each cell and APs are recorded at specific locations.

**Correlation**	**Stimulated at all cells, Recorded at:**			
	**Centroid**	**Surface**	**Edge**	**Vertex**
Height vs. Width	−0.88	−0.94	−0.86	−0.83
*C*_25,10_ vs. AHP	−0.88	−0.74	−0.69	−0.66
*C*_25,10_ vs. ADP	0.92	0.79	0.74	0.7
AHP vs. ADP	−1.00	−1.00	−1.00	−1.00

It can be observed that the correlations obtained for Convexity vs. AHP and Convexity vs. ADP, are contrary to those evaluated from the experimental studies. Such a contradiction could stem from the lack of propagated APs in the bundled detrusor and the abundance of the same in the simulated undifferentiated syncytium. This was tested using another simulation setting, in which all the cells in the syncytium were stimulated successively and only the APs elicited at the stimulated cells were recorded. This ensured that the propagated APs were eliminated from the analysis. The correlation values obtained from these simulations are tabulated in Table 4. It can be noted that the contradicting feature correlations, i.e., Convexity vs. AHP and Convexity vs. ADP, are now flipped, and match the correlation trends obtained from the experimental APs.

**Table 4:** Correlation between AP features from simulations for syncytia of varying sizes, when stimulus is applied successively at each cell and APs are recorded only from the stimulated cells.

**Correlation**	**Stimulated at all cells, Syncytium Size:**			
	**3-Cube**	**5-Cube**	**7-Cube**	**15-Cube**
Height vs. Width	−1.00	−0.98	−0.99	−0.99
*C*_25,10_ vs. AHP	0.81	0.73	0.67	0.65
*C*_25,10_ vs. ADP	−0.95	−0.93	−0.94	−0.97
AHP vs. ADP	−0.90	−0.83	−0.75	−0.71

**Figure 8:**
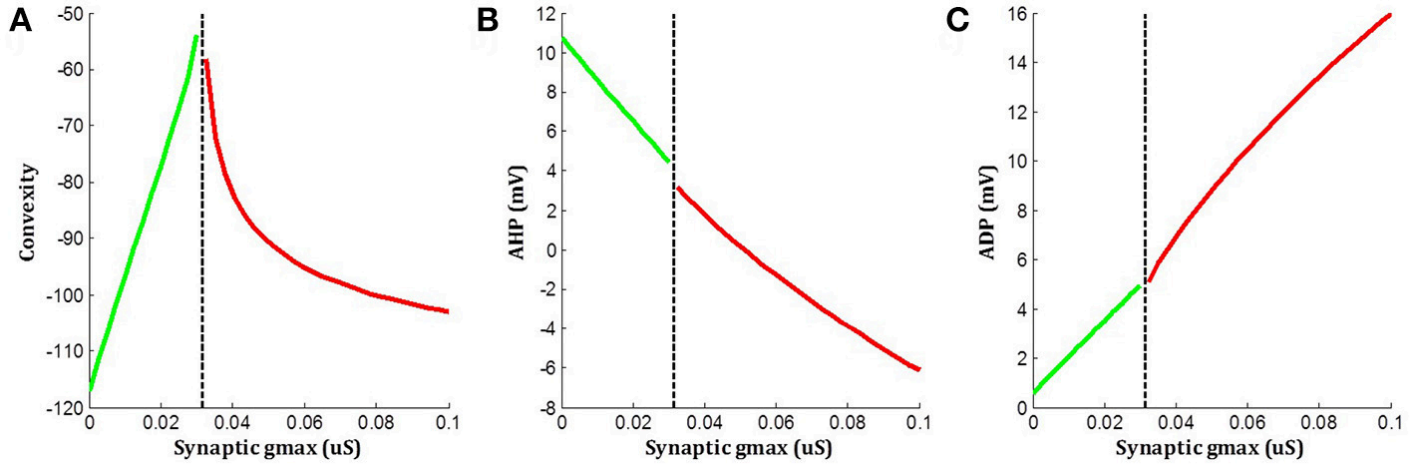
Variations in **(A)** convexity, **(B)** AHP, and **(C)** ADP with change in underlying sEJP. Red traces show data for APs elicited at the centroid cell by a range of synaptic conductances. Green traces show data for APs propagated to the centroid cell in the presence of an underlying sEJP of varying synaptic conductances. The maximum and minimum value of synaptic conductance for the green and red traces, respectively, corresponds to the minimum conductance required for eliciting an AP at the centroid cell.

The above simulations indicated that when an underlying sEJP was present, the correlation of convexity and ADP was negative, while that of convexity and AHP was positive. These trends were flipped around in the case of propagated APs, which had no underlying sEJPs. The variation in the three features—convexity, AHP, and ADP—for different amplitudes of underlying sEJP are shown in Figure 8. The red traces show data for APs elicited at the centroid cell by a range of synaptic conductances. The green traces show data for APs elicited at a vertex cell, and subsequently propagated to the centroid cell in the presence of an underlying sEJP of varying synaptic conductance levels. As the correlation trends are opposite in these two cases, these can serve as indicators for determining the nature of the APs origin.

The observation of the discrepancies between the features of experimentally obtained and simulated APs indicate that the model does not accurately reflect the true biophysical environment. One aspect where the real detrusor syncytium and the computational model employed here could differ is the intensity of the gap junctional coupling between the cells. In order to investigate the effect of variations in gap-junction coupling on the correlation values between the AP features, another set of simulations were conducted.

**Figure 9:**
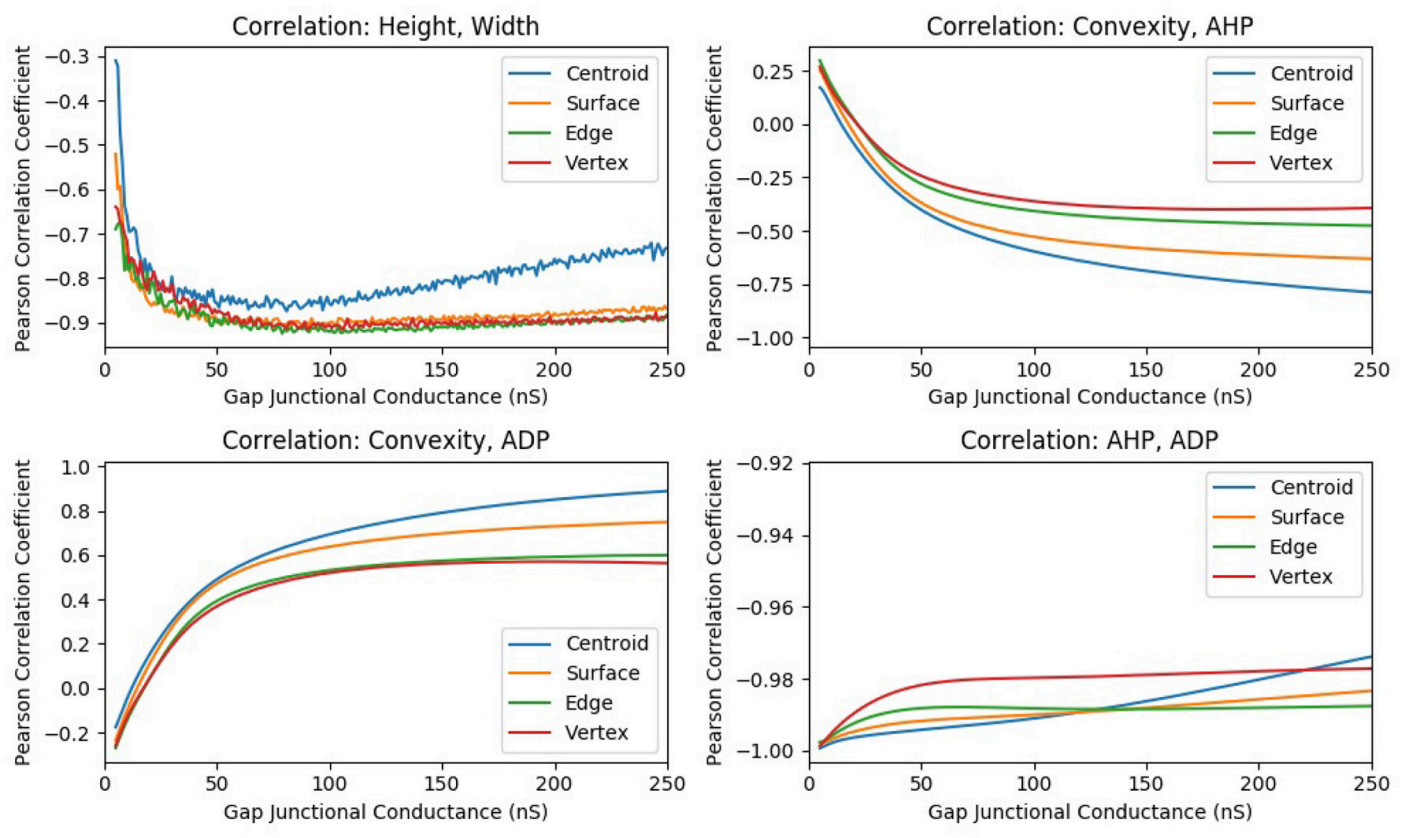
The change in correlation coefficient between various parameters with respect to the gap junction coupling between the smooth muscle cells in the syncytium. The intensity of suprathreshold stimulus was maintained constant across all simulations.

In one such study, the strength of gap junctional conductance, *G*_*gap*_, was varied in the range of 5–250 nS while maintaining the homogeneity of the syncytium. This range is a subset of the experimentally reported range of *G*_*gap*_ values. For each conductance level, all the cells in the syncytium were sequentially stimulated via synaptic input of sufficiently large intensity so as to elicit an AP at every cell in the syncytium, for all values of *G*_*gap*_ in the range. Thus, the stimulus intensity was maintained constant throughout this set of simulations. For each value of *G*_*gap*_, the features of the APs observed at specific locations of the syncytium were evaluated and their correlation coefficients were determined. The plots of the correlation coefficients obtained for each of the *G*_*gap*_ values are given in Figure 9. It can be observed from the figure that the panels AHP vs. ADP and Height vs. Width shows strong correlations throughout the range of *G*_*gap*_ values whereas the panels involving convexity (*C*_*X, Y*_ vs. AHP; *C*_*X, Y*_ vs. ADP) show significant variations in correlation values, even displaying a reversal of the sign of correlation coefficient for very low values of *G*_*gap*_.

Another study involving a heterogeneous variation in the gap junction coupling is described in the section S2 of the supplementary document. A summary of all simulation experiments conducted in this work is tabulated in Table 5.

**Table 5:** Summary of the various simulation scenarios employed.

**Table 2**	*Varied parameter:*	Size of the syncytium
	*Stimulated at:*	Centroidal cell
	*Recorded from:*	All cells
	*Data analysis:*	Collated by syncytial size
	*Number of sims:*	4; one per syncytial size
**Table 3**	*Varied parameter:*	Recording site
	*Stimulated at:*	Each cell; one at a time
	*Recorded from:*	Specific locations
	*Data analysis:*	Collated by location
	*Number of sims:*	125; one per stimulated cell
**Table 4**	*Varied parameter:*	Recording site, Size of the syncytium
	*Stimulated at:*	Each cell; one at a time
	*Recorded from:*	Stimulated cell
	*Data analysis:*	Collated by syncytial size
	*Number of sims:*	3870; one per stimulated cell per syncytial size
**Figure 9**	*Varied parameter:*	Strength of gap junctional coupling
	*Stimulated at:*	Each cell; one at a time
	*Recorded from:*	Specific locations
	*Data analysis:*	Collated by location per coupling strength
	*Number of sims:*	30750: one per stimulated cell per coupling strength
**Table S1**	*Varied parameter:*	Number of coupled cells
	*Stimulated at:*	Each cell; one at a time
	*Recorded from:*	Stimulated cell
	*Data analysis:*	Collated by configuration of coupled cells
	*Number of sims:*	750; one per simulated cell per coupling configuration

## 4. Discussion

In the current study we have restricted our focus to spontaneous action potentials. The origin of these signals is still open to debate. Previous studies have indicated that most of the APs observed under resting conditions are driven by sEJPs (Young et al., 2008). However, there are other studies which suggest a myogenic origin for these signals (Hashitani and Brading, 2003). Our understanding is that regular/periodic action potential firing is likely to be myogenic in origin, perhaps generated by pacemaking cells electrically coupled to the smooth muscle cells, whereas asynchronous/stochastic action potential firing may be driven by spatio-temporally random ATP release from autonomic nerve terminals. Studies excluding the possibility of a neural basis for these signals tested the effects of drugs such as tetrodotoxin and atropine, and found that the signals were not inhibited (Hashitani et al., 2001). Tetrodotoxin inhibits nerve AP-based, but not spontaneous, neurotransmitter release and atropine blocks the ACh receptors on the post-synaptic side. As the varicosities in the detrusor co-transmit both ATP and ACh, it is possible that the spontaneous release of neurotransmitters, evidenced by the presence of sEJPs, is responsible for eliciting the spontaneous action potentials.

Action potentials are commonly referred to as all-or-none signals as they are found to propagate without any change in amplitude or shape, setting them apart from graded potentials such as junction potentials and electrotonic potentials. But this does not always hold true, and fails under certain conditions. It is reported that in long continuous axons or muscle fibers, differences are observed in the AP profiles when recorded at varying distances from the site of initiation (Fatt and Katz, 1951). They also demonstrated that with increase in the distance from the site of stimulus (i.e., the end-plate), the height of the AP increased and the AP foot progressively transformed from being convex-upwards to concave-upwards. Also, at recordings taken closer to the end-plate, a “hump” is seen following the peak indicating the underlying end plate potential (EPP; skeletal muscle equivalent of sEJP) due to the continued action of the neurotransmitter.

The AP, though considered an active signal generated by the membrane, has passive components as well. The part of the AP starting from the instant at which the membrane voltage reaches threshold, to the end of the repolarization phase is mostly governed by the properties of the active ion channels present in the cell membrane. However, the rest of the AP, namely the foot and the end of the tail—shown by the shaded regions in Figure 1A—are predominantly influenced by the passive properties of the cell membrane. Our hypothesis predicts strong correlations between the Convexity, AHP, and ADP of the APs. From Figure 1, it can be observed that the taller the underlying sEJP, the smaller the convexity. A tall sEJP would result in a more depolarized AP tail as it has a more lasting influence. This results in a positive correlation between convexity and AHP. The ADP, according to the hypothesis, is created by the underlying sEJP and would not be seen in the propagated APs. The ADP is measured in the direction opposite to AHP. Hence when the AP tail rises, the ADP increases and vice versa. So a negative correlation is expected between the convexity and the ADP and also between the AHP and ADP. The strong correlations obtained between convexity, AHP, and ADP from experimental recordings are in accordance with expectations. This strongly supports the hypothesis that there is an sEJP underlying these APs.

But in simulations using our computational model of the smooth muscle syncytium, a strong negative correlation was found between the convexity and AHP, and a strong positive correlation between convexity and ADP. This is caused by the fact that in the simulations, all the APs measured, except for the one at the site of stimulus, were propagated APs. As these have no underlying sEJP, the convexity would be low, with a concave upwards AP foot, while the AHP would be maximum—resulting in a negative correlation. In addition, these propagated signals would not exhibit an ADP. Such propagated APs were not frequently observed in our experimental recordings. As explained below and backed by experimental findings, this could be attributed to the pattern of innervation and the size of the syncytium containing the recorded cell. Thus, the nature of correlations between the convexity and ADP/AHP is able to provide an indication of the nature of the APs—whether propagated, or originated at the recorded cell (or in close proximity to it).

Our interpretation is that all the APs observed in the experimental recordings are either locally generated within the cell, by neurotransmitter release from a nearby varicosity, or propagated to it from such a cell in very close vicinity. This release of neurotransmitter would not be nerve-evoked, but arise from the asynchronous release of neurotransmitter packets from the nerve terminals. This random nature of occurrence of APs is confirmed by the exponential trend of the Inter-Event Interval (IEI) histogram. If such is the case, then to explain the wide variety of shapes observed from the intracellular recordings, and their high frequency of occurrence, it is expected that the cells are densely innervated. Studies have shown that the detrusor tissue is densely innervated (Gabella, 1995; Drake et al., 2000, 2003), with around 3 to 4 varicosities per cell (Daniel et al., 1983), consistent with this idea. To verify the experimental correlation trends computationally, we modified the simulation analysis to only include APs recorded at the site of stimulus. Every cell in the syncytium was successively stimulated and the APs elicited at these cells were recorded. It was found that the correlation trends for convexity with AHP and ADP now flipped to match those observed experimentally, with the other correlation trends remaining similar. For example, in a syncytium of size 5-cube, convexity and AHP had a positive correlation of 0.73, while convexity and ADP had a negative correlation of −0.93 (Table 4).

Also, passive signals decay quickly with distance, and thus they cannot be recorded in distant cells. The sEJPs can be detected only at cells in the immediate vicinity, as reported in our earlier work (Appukuttan et al., 2015b). Therefore, in smaller sized syncytia it is likely that the AP recorded at each cell would be influenced by the passively propagated sEJP to varying extents, whereas in larger syncytia the majority of the cells would be unaffected by the sEJP and exhibit a purely propagated AP. This is exemplified in Table 2 where we can see that the correlation of convexity with AHP and ADP deteriorate with increasing size of the simulated syncytium. In the case of the detrusor, experimental studies suggest that the bundles are quite small in size (Hashitani et al., 2001; Neuhaus et al., 2002). Hence it is expected that there is a lack of propagated APs, characterized by their low convexity and high AHP, in the experimental recordings. Correspondingly, in pathology characterized by stronger coupling (Mills et al., 2000) and thus greater electrical reach, there is a likelihood to observe a greater proportion of APs with concave-upwards foot, analogous to propagated APs.

A strong negative correlation exists between the AP height and width. This is also observed in the simulation studies, with the APs having smaller height recorded closer to the site of stimulation. Several factors may contribute to this trend. The site of initiation has to depolarize a larger volume, as every other cell connected to it is at rest, and hence undergoes rapid charge dissipation, resulting in a slower rate of depolarization and a much reduced AP height. This may be further accentuated by the fact that an increased latency in attaining its threshold will result in the inactivation and activation of a higher fraction of Na^+^/Ca^2+^ and K^+^ channels, respectively. In addition, the synaptic conductance at the stimulated cell acts as a shunting pathway, resulting in a lowered input resistance and thereby reduced membrane potential (Fatt and Katz, 1951).

With the help of above inferences, it is now potentially feasible to characterize the cell environment by evaluating the sAP profile. For example, large amplitudes suggest that the cell is located near the periphery of the DSM bundle, whereas smaller amplitudes are indicators of being located near the center and being well connected with the neighboring cells. Presence of ADP along with foot convexity would indicate that the cell receives neurotransmitter input from a nearby varicosity. On the contrary, if the sAP does not exhibit foot convexity and ADP, it implies that the cell exhibits a propagating sAP, which in turn reveals that the cell is part of a larger bundle and being well-coupled to the neighboring cells. From the width of the stimulus evoked sAP, the gap junction connectivity can be estimated. Cells well-coupled to neighboring cells would exhibit sAPs having a larger width, and vice versa. The above are preliminary interpretations, and would need to be further refined following enhancements to the model as proposed ahead.

The simulation studies involving modification of gap junctional conductance of the computational model helped reinforce our reasoning for the observed discrepancy between the correlation trends observed in the experimental and simulation studies. This is elaborated further in section S3 of the supplementary document. One of the objectives of exploring a wide range of values is to explore the effect of variations in such parameters over a wide range of biologically plausible settings in order to study the effects on bladder functioning both in physiology and disease. The range of gap junctional coupling values explored here cover a broad range of experimentally reported values. Christ et al. (2003) have reported a range between 5 and 20 nS for the rat bladder. Other studies have reported intercellular coupling strengths in biological tissues to be as high as 250 ns (Maurer and Weingart, 1987; Lin and Veenstra, 2004), and even higher (Kameyama, 1983). It may be inferred from the simulations that the non-monotonic variation of the convexity parameter with respect to the amplitude of the underlying sEJP causes a reversal of correlation values. This indicates that the “convexity” parameter, on its own, may not be an accurate indicator for the underlying sEJP amplitude.

### 4.1. Limitations of the study

While the results presented here reinforce our hypothesis for the AP generation mechanism, and is capable of explaining several of the observed AP shapes (Figure 4), however, we believe that other factors and mechanisms could also be involved in influencing AP shapes, such as the pacemaker type AP (Figure 4H) and APs with a prolonged after-hyperpolarization phase (Figure 4E). It is therefore relevant to reiterate here that we do not seek to dismiss other possible factors as contributing to AP diversity, such as variations in ionic channel compositions and/or other mechanisms.

As stated earlier, we have opted for a simpler and well-understood AP model in the current study. The HH model employed here basically considers two ionic species (Na^+^ and K^+^) and defines a voltage-gated channel mechanism for each. It should be borne in mind that the detrusor smooth muscle system involves several ionic species with a wide ensemble of ionic channels. These include both voltage-gated (e.g., L-type Ca^2+^, T-type Ca^2+^) as well as ligand-gated (e.g., Ca^2+^ activated BK, IK, SK) channels. Moreover, the intracellular Ca^2+^ dynamics holds potential to exert a consequential influence over the functioning of these channels. Thus, there potentially exists an even larger scope for diversity in the AP profiles observed in the detrusor.

### 4.2. Extensions to the model

With our current level of understanding, we believe it is inconceivable to predict the relative contributions of the various factors toward the observed AP profile. We believe it is necessary to reduce these down into simpler intelligible units and identify their individual contributions, before attempting to explore the interplay between the various mechanisms. The present study sheds light on one such mechanism, namely the syncytial nature of the detrusor smooth muscle.

The ensemble of ion channels and associated calcium dynamics appropriate to reproduce the detrusor AP is another such mechanism. Such a model has not yet been satisfactorily developed. In the past, a model for uterine smooth muscle AP has been reported (Tong et al., 2011) by demonstrating its electrical response in a single cell environment. Some of us are currently involved in developing a similar detrusor specific AP model, which when embedded in the current syncytial model would render it biologically more accurate.

The current model could be further elaborated by endowing it with spatial heterogeneity. Such heterogeneity is known to exist physiologically, as evidenced by the presence of smooth muscle bundles (Brading, 1997), and varying degrees of electrical connectivity between cells (Bramich and Brading, 1996). Certain outcomes of heterogeneous coupling within the syncytium have been presented in the supplementary document (section S2). The existence of cell bundles that differ in size constitutes another source of heterogeneity. Variations of such nature can be relatively easily accommodated in the syncytial model employed here, with the capacity to closely evaluate the outcomes, but this requires awaiting the availability of relevant experimental data indicating such heterogeneity.

### 4.3. Conclusion

Our study has demonstrated that a homogeneous syncytium consisting of smooth muscle cells with identical membrane properties can produce APs of varying shapes. The variations in AP features obtained through simulations were similar in nature to those observed in the experimental APs. Closer examination of the differences between the experimental and simulated AP features and their correlations provided insight into the biophysical environment of the detrusor syncytium, such as the size of the bundles, the density of innervation, and the strength of gap junction coupling between cells. It could also be inferred that the fraction of the propagated action potentials is very low in the detrusor.

The work presented here provides a different approach to exploring the properties of a smooth muscle syncytium. In view of the diversity in AP profiles observed even from a single cell, the findings reported here hold much significance. Since it has now been demonstrated that the variations in AP shapes observed in the syncytium are influenced by syncytial properties, one can analyze the intracellular recordings from individual cells and potentially infer the syncytial environment in which that cell is located. By classifying the cells based on the variations displayed in their electrical activities, an understanding of different aspects of the syncytium could be obtained. Further, this mapping between the AP shape and the syncytial properties could also be used to delineate the syncytial changes during disease and provide a better understanding of pathological conditions.

## Ethics statement

The Institutional Animal Care and Use Committee (IACUC) covering the Department of Pharmacology, University of Oxford, approved and had oversight of all animal experiments. All experiments were carried out during or before 2009, and were hence approved under Animals (Scientific Procedures) Act 1986, but are also consistent with both UK Animals (Scientific Procedures) Act (2013) and European Communities Council Directive 2010/63/EU.

## Author contributions

SA designed the computational studies, performed the simulations, and wrote the first draft of the manuscript. MP designed the analysis of experimental data, carried out the statistical analysis, and contributed in writing the manuscript. JY and KB performed the electrophysiological recordings and helped revise the manuscript. RM contributed toward the interpretation of the results and in revising the manuscript.

### Conflict of interest statement

The authors declare that the research was conducted in the absence of any commercial or financial relationships that could be construed as a potential conflict of interest.
