# Supplementary material for: Investigation of the Syncytial Nature of Detrusor Smooth Muscle as a Determinant of Action Potential Shape
Source: Front Physiol. 2018 Sep 20;9:1300. doi: 10.3389/fphys.2018.01300 (PMC6158746; doi:10.3389/fphys.2018.01300)
Supplement: Supplementary file 1 [file Data_Sheet_1.pdf]

Supplementary Document: Investigation of the Syncytial Nature of Detrusor Smooth Muscle as a Determinant of Action Potential Shape

Shailesh Appukuttan [1,#], Mithun Padmakumar [1,#], John S. Young [2], Keith L. Brain [3,4] and Rohit Manchanda [1,*]

[1] *Computational Neurophysiology Lab, Department of Biosciences and Bioengineering, Indian Institute of Technology Bombay, Mumbai , India*
[2] *School of Pharmacy and Biomedical Sciences, University of Portsmouth, Portsmouth, UK*
[3] *Institute of Clinical Sciences, College of Medical and Dental Sciences, University of Birmingham, Birmingham, UK*
[4] *Christ Church, University of Oxford, UK*
[#] *These authors contributed equally to this work*

Correspondence*:
Prof. Rohit Manchanda, Computational NeuroPhysiology Lab, Department of Biosciences and Bioengineering, IIT Bombay, Powai, Mumbai - 400076, India;
rmanch@iitb.ac.in

S1 MODEL DEVELOPMENT

An overview of the development of the model has been described in the manuscript. Here, we present some additional details. Table S1 presents the parameters, and their values, employed in constructing the model. Fig. S1 illustrates the intercellular coupling of cells and their arrangement in space to form a three dimensional syncytium. For a more detailed description of the model development, the reader is referred to Appukuttan et al. (2015).

Table S1. Biophysical properties of the individual cells and the gap junctions coupling them

Parameter	Value
Cell length	200 μm
Cell diameter	6 μm
Number of segments/cell	51
Axial resistivity	183 Ωcm
Resting potential	-65 mV
Membrane capacitance	1 μF/cm^2
Gap junctional resistance	30.6 MΩ

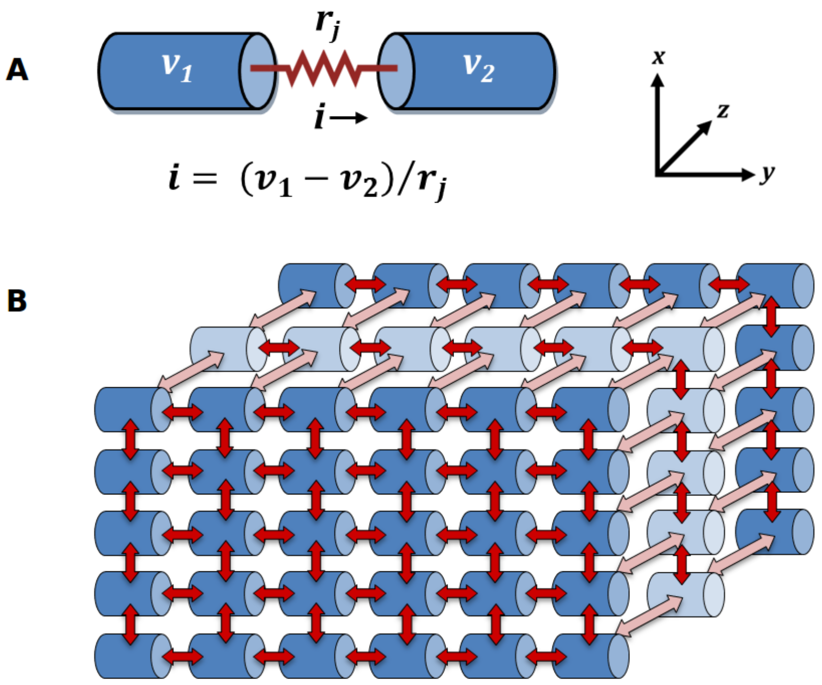

Figure S1. Conceptual representation of the construction of the syncytium: **(A)** gap junctions between cells were modeled as bi-directional passive resistive pathways. The magnitude and direction of current flowing across the gap junctional was determined by the potential gradient between the coupled cells, and the strength of gap junctional coupling, **(B)** arrangement of cells in a cubic lattice layout to form a three-dimensional syncytium. Source: Appukuttan et al. (2017)

S2 SIMULATION OF HETEROGENEOUSLY COUPLED SYNCYTIUM

All the earlier simulations involved homogeneous coupling within the syncytium. That is, all cell pairs were linked identically along each axis. Even in simulations where the gap junctional coupling strength was varied, this was carried out uniformly across all gap junctions. Here, the homogeneity of the syncytium was compromised by selectively removing the gap-junction coupling between certain pairs of cells. Two variants of heterogeneous coupling was tested within a syncytium of size 5-cube. It would be useful to note that a 5-cube syncytium in our model has a total of 300 cell pairs; 100 along each axis.

- **Probability per gap junction:** The decision to form a gap junction between each cell pair was determined based on a pre-defined probability. The correlation trends for this protocol have been presented in Table S2.
- **Number of coupled cells:** In this variant, the total number of gap junctions along each axis was first determined based on the specified fraction. The specific cell pairs that were coupled was then selected from a uniform distribution. The correlation trends for this protocol have been presented in Table S3.

The synaptic stimulus was applied to each of the cells in succession. Correlation trends were evaluated only for the stimulated cell and not for specific locations (e.g. centroid, vertex) as in a heterogeneously coupled syncytium the locations do not have the same connotation as in a homogeneously coupled syncytium. It is worth mentioning that the synaptic time constant had to be halved (from 10 ms to 5 ms) for these simulations; still remaining within the experimentally recorded range of 5–89 ms (Young

Table S2. Correlation between AP features from simulations for a syncytium of size 5-cube, for varying levels of gap junction coupling probabilities. Stimulus is applied successively at each cell and APs are recorded from the stimulated cells.

Correlation	Stimulated at all cells, Gap junction probability					
	50%	60%	70%	80%	90%	100%
Height vs Width	-0.99	-0.99	-0.98	-0.98	-0.99	-1.00
$C_{25,10}$ vs AHP	0.74	0.83	0.87	0.90	0.93	0.94
$C_{25,10}$ vs ADP	-0.94	-0.89	-0.86	-0.85	-0.85	-0.89
AHP vs ADP	-0.62	-0.73	-0.80	-0.82	-0.88	-0.93

Table S3. Correlation between AP features from simulations for a syncytium of size 5-cube, for varying fractions of coupled cell pairs. Stimulus is applied successively at each cell and APs are recorded from the stimulated cells.

Correlation	Stimulated at all cells; % Coupled cell pairs					
	50%	60%	70%	80%	90%	100%
Height vs Width	-0.98	-0.99	-0.99	-0.99	-0.98	-0.99
C25,10 vs AHP	0.68	0.79	0.88	0.89	0.91	0.94
C25,10 vs ADP	-0.93	-0.88	-0.88	-0.82	-0.87	-0.89
AHP vs ADP	-0.57	-0.69	-0.82	-0.83	-0.88	-0.93

et al., 2008). This was done to avoid a second AP arising out of a single stimulus owing to prolonged depolarization, and the elevated excitability of cells in a sparsely coupled syncytium.

S3 DISCUSSION ON IDENTICAL STIMULATION IN HOMOGENEOUS SYNCYTIUM

The variation in the correlation trends with respect to the gap-junction conductance, G_{gap}, is shown in main text of the manuscript as Fig. 9. It was observed that the correlations involving convexity values ($C_{X,Y}$ vs AHP and $C_{X,Y}$ vs ADP) shows a drastic variation, even changing the sign of the correlation coefficient. An attempt is made here to investigate the reason behind such an observation.

The individual feature values obtained from all 125 cells for a specific G_{gap} value were taken from different locations - (i) centroid, (ii) surface, (iii) edge, and (iv) vertex. The mean values of features were evaluated and were plotted against G_{gap} for every G_{gap} value in the study range. The plots thus obtained are shown in Fig. S2. Here it can be seen that the mean values shown by the convexity values have a highly non-monotonic trend.

It is hypothesized that the AP convexity measured by the parameter $C_{X,Y}$ indicates the amplitude of the sEJP underlying the AP. Here, while increasing G_{gap} for a same stimulus intensity, the input resistance of the syncytium decreases which in turn reduces the sEJP amplitudes. If the above hypothesis was true, the $C_{X,Y}$ value should have shown a monotonous reduction. From the trend of the $C_{X,Y}$ values shown in Fig. S2, it is not the case which contradicts the hypothesis. This non-monotonic behavior of the $C_{X,Y}$ is the reason behind the drastic variation of correlation values in Fig. 9 where convexity feature was involved.

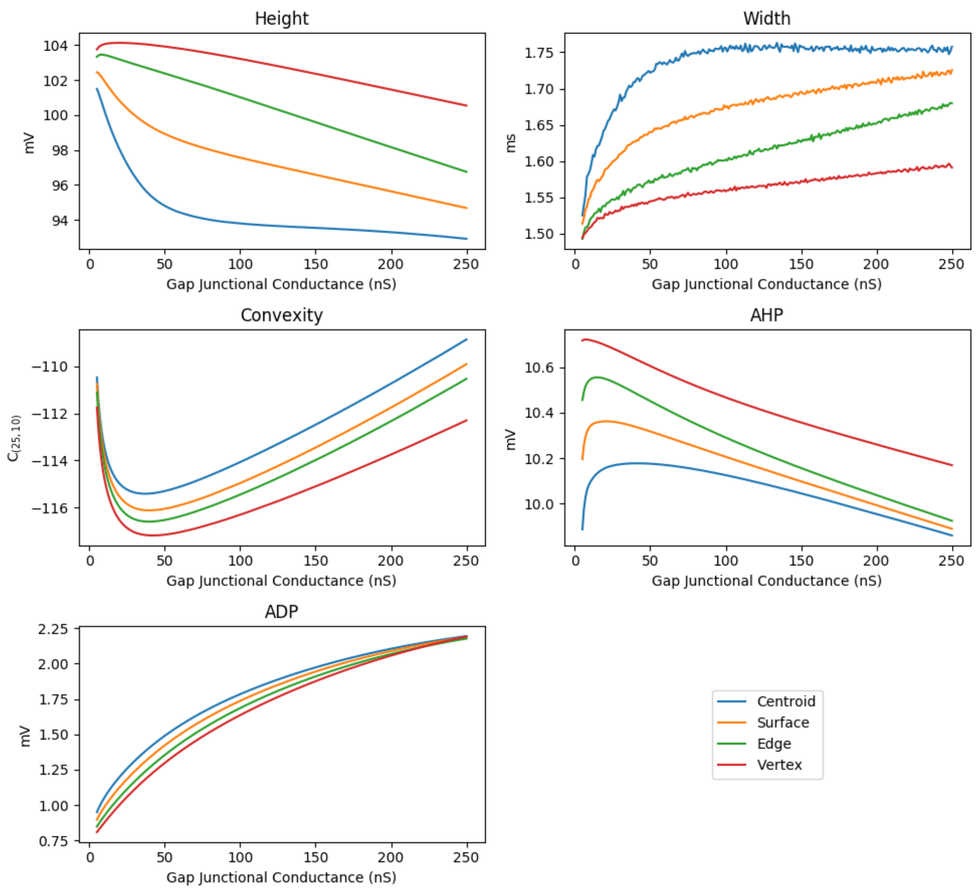

Figure S2. Average feature values obtained for APs observed from specific locations in the homogeneous syncytium, with respect to variation in gap junctional conductance (G_{gap}). For a single G_{gap} value, each of the 125 cells present in the 5-cube syncytium were stimulated and resulting APs observed from specific locations indicated in the legend. The features were evaluated from the observed APs and the mean values were plotted. Note the highly non-monotonic characteristics of the 'Convexity' feature.

REFERENCES

Appukuttan, S., Brain, K., and Manchanda, R. (2017). Investigation of action potential propagation in a syncytium. *Biomedical Research Journal* 4, 102–115

Appukuttan, S., Brain, K. L., and Manchanda, R. (2015). A computational model of urinary bladder smooth muscle syncytium. *Journal of computational neuroscience* 38, 167–187

Young, J. S., Meng, E., Cunnane, T. C., and Brain, K. L. (2008). Spontaneous purinergic neurotransmission in the mouse urinary bladder. *The Journal of Physiology* 586, 5743–5755
